# Multi-Step Ubiquitin Decoding Mechanism for Proteasomal Degradation

**DOI:** 10.3390/ph13060128

**Published:** 2020-06-23

**Authors:** Hikaru Tsuchiya, Akinori Endo, Yasushi Saeki

**Affiliations:** Protein Metabolism Project, Tokyo Metropolitan Institute of Medical Science, Tokyo 156-8506, Japan; tsuchiya-hk@igakuken.or.jp (H.T.); endo-ak@igakuken.or.jp (A.E.)

**Keywords:** proteasome, protein degradation, ubiquitin, proteasomal ubiquitin receptors, p97/VCP, liquid–liquid phase separation

## Abstract

The 26S proteasome is a 2.5-MDa protease complex responsible for the selective and ATP-dependent degradation of ubiquitylated proteins in eukaryotic cells. Proteasome-mediated protein degradation accounts for ~70% of all cellular proteolysis under basal conditions, and thereby any dysfunction can lead to drastic changes in cell homeostasis. A major function of ubiquitylation is to target proteins for proteasomal degradation. Accompanied by deciphering the structural diversity of ubiquitin chains with eight linkages and chain lengths, the ubiquitin code for proteasomal degradation has been expanding beyond the best-characterized Lys48-linked ubiquitin chains. Whereas polyubiquitylated proteins can be directly recognized by the proteasome, in several cases, these proteins need to be extracted or segregated by the conserved ATPases associated with diverse cellular activities (AAA)-family ATPase p97/valosin-containing protein (VCP) complex and escorted to the proteasome by ubiquitin-like (UBL)–ubiquitin associated (UBA) proteins; these are called substrate-shuttling factors. Furthermore, proteasomes are highly mobile and are appropriately spatiotemporally regulated in response to different cellular environments and stresses. In this review, we highlight an emerging key link between p97, shuttling factors, and proteasome for efficient proteasomal degradation. We also present evidence that proteasome-containing nuclear foci form by liquid–liquid phase separation under acute hyperosmotic stress.

## 1. Introduction

The ubiquitin–proteasome system is the primary degradation system in eukaryotic cells. The 26S proteasome is a huge enzyme complex composed of the 20S core particle (CP; also known as 20S proteasome) and one or two 19S regulatory particles (RPs). There are 33 distinct proteins, with a total of 66 subunits, which constitute 26S proteasome. The CP has a barrel-shaped structure that possesses two sets of three proteolytic active sites inside the cavity. The RP recognizes the polyubiquitin chains via intrinsic ubiquitin receptors (Rpn10, Rpn13, and Rpn1), unfolds the substrate proteins, and translocates them into the catalytic CP [1]. Besides the RP, the proteasome has alternative proteasome activators, such as PA28α/β (PSME1/2), PA28γ (PSME3), and PA200 (PSME4), which are involved in ubiquitin-independent protein degradation (reviewed by Kish–Trier et al.) [2]. Through the degradation of substrates, the proteasome plays an essential role in most cellular functions, such as cell cycle progression, transcription, signal transduction, and protein homeostasis [3]. In addition to the constitutive subunits, the proteasome has multiple transiently-associated proteins called proteasome-interacting proteins. Among them, substrate shuttling factors, ubiquitin ligases, and deubiquitylating enzymes modulate proteasome functions. It is now clear that ubiquitin-selective AAA-ATPase p97 and substrate shuttling factors like RAD23 and UBQLNs act to select and deliver ubiquitylated substrates upstream of the proteasome [4]. In this review, we describe the recent progress in our research on factors upstream of the proteasome, including substrate shuttling factors and p97, the selectivity of the ubiquitin chain for proteasomal degradation, and recently identified nuclear proteasome droplets that are formed under the hyperosmotic stress triggered by multivalent interactions between RAD23B and ubiquitylated proteins to be degraded. These findings underline the multi-step regulation of proteasome responding to the cellular environment.

## 2. Ubiquitin-Binding Proteins Related to the Proteasome: Proteasomal Ubiquitin Receptors, UBL–UBA Proteins, and p97

### 2.1. Proteasomal Ubiquitin Receptors (Rpn10, Rpn13, and Rpn1)

**Figure 1:**
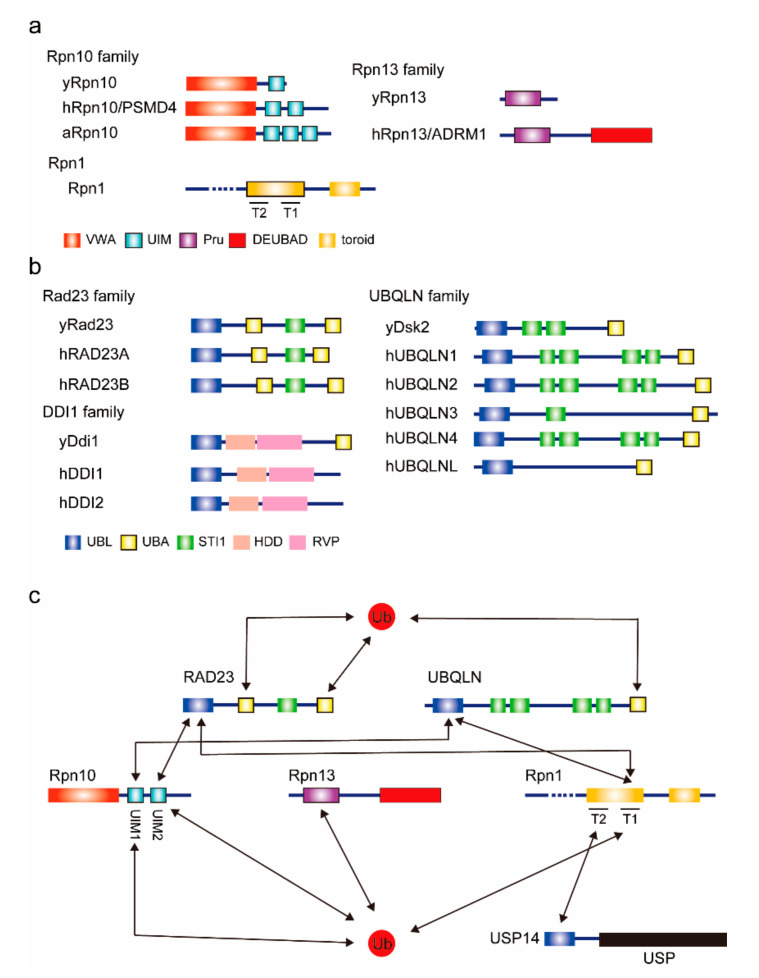
Proteasomal ubiquitin receptors. (**a**) Domain architectures of intrinsic proteasomal ubiquitin receptors. Known ubiquitin and ubiquitin-like (UBL) domain-binding domains are indicated by thick-bordered boxes. The prefix “h” indicates the human protein; “y” indicates the yeast protein; “a” indicates the arabidopsis protein. VWA, von Willebrand factor A. UIM, ubiquitin-interacting motif. Pru, Pleckstrin-like receptor for ubiquitin. DEUBAD, deubiquitinase adaptor. (**b**) Domain architectures of extrinsic ubiquitin receptors. UBL, ubiquitin-like. UBA, ubiquitin associated. HDD, helical domain of Ddi1. RVP, predicted retroviral protease-like. (**c**) The interaction map for ubiquitin and ubiquitin receptors. Known interactions are indicated by arrows. USP, ubiquitin-specific protease.

In proteasomal degradation, the recognition of ubiquitin chains is one of the most crucial steps. The proteasome has three defined intrinsic ubiquitin receptors, namely Rpn10, Rpn13, and Rpn1, that recognize ubiquitylated substrates on the proteasome. These ubiquitin receptors can also bind ubiquitin-like (UBL) domains and are therefore also known as UBL domain receptors (Figure 1).

Rpn10 (also known as S5a/PSMD4) is composed of an N-terminal von Willebrand factor A (VWA) domain and C-terminal ubiquitin-interacting motif (UIM) domains. The VWA domain interacts with the proteasome and the UIM domain binds to ubiquitin chains. The UIM domain consists of a single alpha helix. Yeast Rpn10 has one UIM domain, but human and plant Rpn10 have two and three UIMs, respectively [5]. The additional UIM domain is thought to be the binding site for UBL domain-containing proteins. The UBL domain in RAD23A preferentially binds to UIM-2 in Rpn10 [6], whereas the UBL domain in UBQLN2 exhibits a 25-fold stronger affinity for the N-terminal UIM-1 over UIM-2 in hRpn10 [7]. In plants, UIM-2 specifically interacts with ATG8 for the autophagic turnover of the proteasome, or proteaphagy [8]. Interestingly, recent studies have suggested that the UBL domains in both RAD23 and UBQLN can activate the mammalian proteasome even though they bind to different UIMs in Rpn10 [9,10]. It remains unclear how these UIM domains are coordinated as a platform for ubiquitin chains and UBL domain-containing proteins. Mice lacking all the UIMs in Rpn10 exhibit embryonic lethality, suggesting the importance of UIMs in Rpn10 [11].

Rpn13/ADRM1 binds ubiquitin via a conserved antiparallel β-sheet region, termed the Pru domain (Pleckstrin-like receptor for ubiquitin), which itself binds to Lys48-linked diubiquitin with an affinity of ~90 nM [12]. Rpn13 Pru also binds to UBL domain-containing proteins. NMR analysis revealed that Rpn13 directly binds to RAD23A and UBQLN2, and the Kd value of Rpn13 Pru–RAD23A UBL domain is about 36 μM [12]. Rpn13 also binds to the Uch37/UCHL5 deubiquitylating enzyme via its carboxy-terminal domain, called the deubiquitinase adapter (DEUBAD) domain. Curiously, Rpn13 in budding yeast lacks both the DEUBAD domain and Uch37 [13]. By trimming the ubiquitin chains, Uch37 may remodel ubiquitin chains on ubiquitin receptors.

Rpn1 is a large proteasome subunit composed of 11 repeats of 30 to 40 residues, known as proteasome/cyclosome (PC) repeats, each of which forms a helix-turn-helix hairpin [14,15]. These PC repeats form a closed toroid domain consisting of T1 and T2 sites. T1 binds to both ubiquitin and the RAD23 UBL domain, and T2 recognizes the UBL domain of deubiquitylating enzyme Ubp6/USP14 [16]. Thus, the toroid domain in Rpn1 serves as the receptor not only for ubiquitylated proteins but also for shuttling factors and deubiquitylating enzymes.

It has been suggested that, to a certain extent, these ubiquitin receptors have functional redundancy in the ubiquitin recognition of the proteasome. Single gene mutation of the ubiquitin- and UBL domain-binding domains of these ubiquitin receptors is permissive in yeast. In addition, yeast cells that lack all three ubiquitin-binding sites are still viable, although they exhibit amino-acid analog sensitivity [16]. Using a well-defined model substrate and mutant proteasomes, Martinez–Fonts et al. elegantly demonstrated that Rpn10 is a primary receptor for Lys48-linked ubiquitylated substrates, and Rpn1 and Rpn13 are co-receptors for multi-ubiquitylated substrates [17]. Moreover, a recent study showed that Rpn1 binds Lys11/Lys48 branched ubiquitin chains more efficiently than Lys48-linked homotypic chains [18]. Thus, three receptors provide a versatile platform for various ubiquitin chain topologies. In mice, the liver-specific deletion of either RPN10 or RPN13 exhibited modest tissue impairment, but the simultaneous loss of both RPN10 and RPN13 caused severe liver injury accompanied by a massive accumulation of ubiquitin conjugates and UBL domain-containing proteins on the proteasome [19].

Other subunits, such as Rpt5 and Rpn15/Sem1/Dss1, were proposed to bind to ubiquitin [20,21]. However, this process is not yet fully understood.

### 2.2. UBL–UBA Proteins

Some ubiquitylated proteins are recognized and degraded by the proteasome with the help of UBL–UBA domain-containing proteins (UBL–UBA proteins). The main members of the UBL–UBA family are Rad23, also known as RAD23A/B in mammals; Dsk2, also known as UBQLN1~4 and L in mammals; and Ddi1/2. The UBL–UBA proteins contain a UBL domain in the N-terminus and one or two UBA domains in the C-terminus. These proteins simultaneously bind to ubiquitin via UBA domains [22,23] and to the proteasome via the UBL domain, and they shuttle ubiquitylated substrates to the proteasome. Like ubiquitin chains, the UBL domain binds to Rpn1, Rpn10, or Rpn13.

Rad23, which is conserved from yeast to humans, contains an N-terminal UBL domain, a stress inducible-1 (STI) domain (Rad4/XPC binding), and two UBA domains. Rad23 was originally identified as a nucleotide excision repair factor that is involved in DNA repair [24], and it is also characterized as a regulatory factor of intracellular protein degradation. The STI domain is also known to bind *N*-glycanase (Png1 in yeast, NGLY1/2 in mammals), suggesting the involvement of the proteasomal degradation of the glycoprotein that has leaked into the cytoplasm [25]. RAD23A and RAD23B in mammals show high homology in their amino acid sequences and therefore they can functionally compensate for one another’s roles in nucleotide excision repair [26]. Despite this functional redundancy of RAD23A and B, only RAD23B-deficient mice exhibited developmental impairment and growth retardation [26]. The susceptibility of each protein’s depletion in mice may be explained by the difference in protein expression levels in their tissues. Indeed, the expression level of RAD23B is 6- to 10-fold higher than that of RAD23A in cultured human cells, suggesting that the expression levels of these proteins are differentially controlled depending on cell or tissue type.

Dsk2 was originally identified, together with Rad23, as an essential factor for the duplication of the microtubule organization center [27]. Dsk2 contains a UBL domain, an STI domain that binds to heat shock proteins, and a UBA domain. Five UBQLN proteins (UBQLN1~4 and L) are encoded in the human genome. UBQLN1, 2, and 4 have some redundant functions [28], whereas UBQLN3 is mainly expressed in the testis, and UBQLN-L lacks the UBA domain. The STI domain of UBQLNs appears to directly recognize transmembrane proteins, and with their associated ubiquitin E3 ligases, UBQLNs target mislocalized transmembrane proteins that fail to be transported to the endoplasmic reticulum (ER) or mitochondria for proteasomal degradation [29,30]. In addition, the STI domain interacts with the disaggregase HSP70–HSP110 and shuttles aggregated proteins to the proteasome [31]. Notably, mutations in the UBQLN2 gene are linked to amyotrophic lateral sclerosis (ALS) and frontotemporal lobar degeneration [32]. UBQLN4 also participates in the export of ubiquitylated proteins from the nucleus to the cytoplasm through association with an nuclear export signal-containing protein, named POST, and may contribute to nuclear protein quality control [33]. Yeast Rad23 and Dsk2 play a redundant role in proteasomal degradation [34,35] and synergistically contribute to shuttling ubiquitylated substrates to the proteasome [4]. However, human RAD23A/B and UBQLN proteins seem to have some specific functions, e.g., RAD23B and UBQLN proteins contribute to droplet formation by the proteasome [36] (described below) and autophagosome [37], respectively.

Ddi1 (also known as DDI1/2 in mammals) remains less understood. Ddi1 has a helical domain of Ddi1 and a predicted retroviral protease-like domain that has a similar amino acid sequence to retroviral aspartyl proteases; for example, those encoded in HIV [38,39]. Interestingly, the UBL domain of Ddi1 can bind not only to the proteasome but also to ubiquitin, as can the UBA domain [40,41]. Interestingly, recent studies have revealed that yeast Ddi1 is a ubiquitin-dependent protease that preferentially recognizes long Lys48-linked ubiquitin chains and cleaves proteasome substrates [42]. In mammals, the cleavage of DDI2 activates an ER-resident Nrf1 transcription factor that regulates the gene expression of proteasome subunits to compensate for proteasome dysfunction [43].

### 2.3. Cdc48/p97/VCP-Ufd1-Npl4 Complex

**Figure 2:**
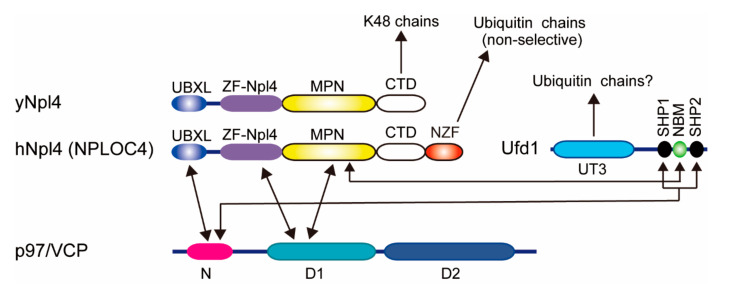
Domain architectures of p97, Ufd1, and Npl4. Known interactions are indicated by arrows. UBXL, ubiquitin regulatory X (UBX)-like. ZF, Zinc finger. MPN, Mpr1/Pad1 N-terminal. CTD, C-terminal domain. NZF, Npl4 zinc finger. NBM, Npl4-binding motif. SHP, small heterodimer partner.

Cdc48 (also known as p97 or VCP in mammals) is a hexameric AAA-type ATPase that is essential for cell survival. p97 functions in many cellular processes, such as ER-associated degradation (ERAD), selective autophagy, and DNA damage responses [44,45]. Multiple different cofactors regulate p97 and generate functional diversity [44]. The Ufd1 (UFD1L in humans)-Npl4 (NPLOC4 in humans) heterodimer is the best-characterized ubiquitin-binding cofactor in proteasomal degradation. Polyubiquitylated proteins which are embedded in membranes or assembled in multi-subunit complexes are extracted by the p97-Ufd1-Npl4 complex using ATP hydrolysis. Human Npl4 consists of an N-terminal ubiquitin-X (UBX)-like domain that binds to p97, a zinc-finger domain (ZF-Npl4), an Mpr1/Pad1 N-terminal (MPN) domain found in JAB1/MPN/Mov34 metalloenzyme (JAMM)-family deubiquitylating enzymes, a C-terminal domain (CTD), and an Npl4 zinc finger (NZF) domain (Figure 2). In mammals, Npl4 binds to ubiquitin via the NZF domain without ubiquitin chain selectivity [46,47,48]. Interestingly, yeast Npl4 lacks the NZF domain, and instead, the ZF-Npl4-MPN-CTD is utilized for ubiquitin binding [4,47]. Since the MPN domain in Npl4 is topologically similar to the catalytic domain of JAMM-family deubiquitylating enzymes, the MPN domain was thought to be the binding site for ubiquitin. However, recent cryo-electron microscopy analysis has shown that the Lys48 ubiquitin chain does not interact with the groove of the MPN domain in yeast Npl4 but rather with the CTD domain and other regions of the MPN domain [49]. The crystal structures of yeast Npl4 in the complex with Lys48-linked diubiquitin revealed that the C-terminal helix and N-terminal loop of CTD mainly interact with the distal and proximal ubiquitin moieties, respectively [50]. Ufd1, rather than ubiquitin, occupies a hydrophobic groove of the MPN domain of Npl4 [50]. Ufd1 also has two short small heterodimer partner (SHP) motifs that bind to p97, and it contains the Ufd1 truncation 3 (UT3) domain that binds to ubiquitin.

### 2.4. The Major Pathway for Proteasomal Degradation

Since the proteasome itself contains the ubiquitin receptors Rpn1, Rpn10, and Rpn13, it was thought that the proteasome directly recognizes ubiquitylated substrates. Nevertheless, the degradation of some ubiquitylated substrates requires the UBL–UBA proteins and p97 prior to recognition by the proteasome [34,35,51,52]. The importance of the UBL–UBA proteins and p97 in proteasomal degradation has been a long-standing question. Quantitative proteomics revealed that proteasome-bound ubiquitin chains were dramatically decreased in *rad23* and *dsk2* knockout cells [4], suggesting that most proteasome substrates are degraded through the Rad23- and Dsk2-dependent pathway. In a p97 temperature-sensitive mutant (*cdc48-3*), ubiquitylated substrates showed significant accumulation on the proteasome. Surprisingly, this accumulation was almost completely abolished by the knockout of *rad23* and *dsk2*, suggesting that Rad23 and Dsk2 are downstream factors of p97, reminiscent of the escort model originally described by Richly et al. [34]. These findings indicated that the p97–Rad23/Dsk2 axis plays a major role in ubiquitin-dependent proteasomal degradation rather than in direct proteasomal recognition and degradation. Recently, two independent groups reported that the p97-Ufd1-Npl4 complex has unfoldase activity that relies on ATP hydrolysis and substrate ubiquitylation [53,54]. Moreover, efficient proteasomal degradation requires the presence of an unstructured region in substrates, which facilitates proteasomal engagement with the substrates and initiates unfolding and degradation [55]. Tomita et al. reported that calpain cleavage exposes a region that is recognized by the proteasome and allows it to initiate degradation efficiently [56]. In this context, the proteasome has difficulty degrading well-folded proteins that lack an unstructured region; therefore, the proteasome needs the assistance of the p97-Ufd1-Npl4 complex [57]. It is assumed that p97 provides the initiation site for the proteasome, and thus the proteasome can efficiently degrade a large number of proteins in cells.

## 3. Ubiquitin Signal for the Proteasome: Linkage Type and Length

### 3.1. Ubiquitin Chain Type Selectivity for Proteasomal Degradation

It is widely known that the tetra-Lys48-linked chain is the primary signal in substrate degradation by the proteasome [58]. Curiously, neither the proteasome itself nor Rad23, Dsk2, or Ddi1 show strict selectivity for Lys48 chains in vitro [59,60]. Recent studies have suggested that various ubiquitin architectures can also target proteins for proteasomal degradation. Multiple mono-ubiquitylation can efficiently induce proteasomal degradation, at least in vitro [61]. Additionally, multiple short chains with Lys11, Lys48, and Lys63 linkages can efficiently induce proteasomal degradation [61,62]. Moreover, in mammalian cells, anaphase-promoting complex/cyclosome (APC/C) substrates modified with Lys11/Lys48 branched chains are degraded more efficiently than those with homogenous Lys48-linked chains [63]. In yeast, Lys29/Lys48 branched chains attached to ubiquitin fusion degradation substrates accelerate proteasomal degradation [64]. Lys63/Lys48 branched chains also trigger proteasomal degradation [65]. To clarify what type of ubiquitin signal is primarily recognized by the proteasome in vivo, we performed a quantitative proteomic analysis of the ubiquitin linkage-type selectivity of major ubiquitin-binding domain (UBD)-containing proteins and the proteasome in yeast [4]. Mass spectrometry-based quantification revealed that the proteasome mainly binds Lys29 and Lys48 chains (about 7% and 90%, respectively), suggesting that these chains are the primary signals in yeast cells. A comparative analysis of the ubiquitin linkage selectivity of 14 major UBD proteins in yeast revealed that the UBL–UBA proteins and all p97 cofactors showed strong preferences for Lys48 linkage, whereas UBD proteins related to endocytosis were associated with K63 linkage. We also found that Npl4 had strict selectivity for the Lys48-linked chains. Given that all upstream factors for the proteasome were highly selective for Lys48- and Lys29-linked chains in vivo, but only Npl4 had Lys48 chain selectivity in vitro, we propose that the p97-Ufd1-Npl4 complex rather than Rad23 and Dsk2 determines the Lys48-linkage selectivity for proteasomal degradation in cells. Notably, although the abundance of Lys11 linkage was estimated to be very low (comprising only 1.3% of the eight different linkages) in our experimental setting, a more recent study demonstrated that Lys11/Lys48-mixed ubiquitin chains are utilized for the proteasomal degradation of misfolded proteins in the cytoplasm [66]. In mammalian cells, mass spectrometry-based quantification revealed that the human proteasome contains relatively high levels of Lys11 and Lys63 linkages, probably derived from Lys11/Lys48 and Lys48/Lys63 branched chains [65], clearly suggesting that proteasome-targeting signals are more complex in higher eukaryotes.

### 3.2. Ubiquitin Chain Length as a Signal for Proteasomal Degradation

Ubiquitin chain length is also an important factor for ubiquitin signaling. Historically, it has been thought that the Lys48 chain is the primary signal for proteasomal degradation. An in vitro analysis revealed that four or more ubiquitin molecules on substrate proteins are sufficient to target proteins to the proteasome [58]. However, a recent study suggested that various chain lengths contribute to substrate degradation in vitro. A single-molecule kinetic analysis showed that the proteasome degrades cyclin B2 with multiple short chains [61,62]. The authors visualized the activity of the APC, a ubiquitin ligase that plays an important role in the cell cycle. The APC/C rapidly attached mono- to tri-ubiquitin molecules to cyclin B2 [61,62]. In addition, mono-ubiquitylated small disordered proteins (<150 amino acids) are sufficiently degraded by the proteasome [67]. Nuclear magnetic resonance studies demonstrated that Rpn10 and Rpn13 simultaneously bind to ubiquitin chains, with a preference for Rpn10 for distal ubiquitin and Rpn13 for proximal ubiquitin [68]. Cryo-electron microscopy analysis revealed that the distance between Rpn10 and Rpn13 is approximately 100 Å, which is the same length as a tetra-ubiquitin chain [69]. One possible scenario is that this distance determines the minimum length for a ubiquitin chain recognized by the proteasome. Despite the fundamental importance of each substrate’s ubiquitin chain length, there have been no methods to determine it, other than through estimation via gel mobility on sodium dodecyl sulfate polyacrylamide gel electrophoresis (SDS-PAGE). To determine the length of ubiquitin chains in biological samples, we designed an approach using trypsin and a ubiquitin chain protector, named “ubiquitin chain protection from trypsinization (Ub-ProT)” [70]. In native conditions, trypsin preferentially cleaves at Arg74 of ubiquitin [71], but substrate-attached chains are protected from trypsinization in the presence of a ubiquitin chain protector such as trypsin resistant tandem ubiquitin-binding entity (TR-TUBE) [70,72]. The Ub-ProT method, using soluble fractions obtained from yeast, revealed that the substrate-attached ubiquitins mainly ranged from monomers to heptamers [70]. Interestingly, the maximal ubiquitin chain length hardly changed as a result of proteasome inhibition, suggesting that the mechanism for regulating the ubiquitin chain length lies upstream of the proteasome in cells. By contrast, in p97 and Npl4 mutants, the global ubiquitin chain length was elongated, suggesting that the recognition and segregation of ubiquitylated substrates by p97-Ufd1-Npl4 are the key steps for determining ubiquitin chain length in cells.

**Figure 3:**
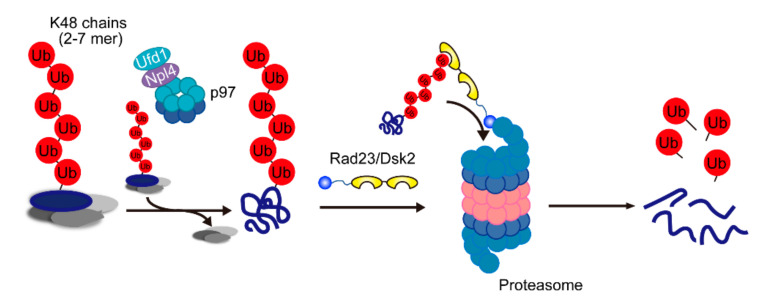
A model of the major route for degradation of proteasomal substrates. Two- to seven-mer Lys48 chains attached on the substrate are recognized by Npl4, and the substrate is segregated by p97. Then, the segregated substrate is transferred to shuttle factors and delivered to the proteasome.

On the basis of these findings, we proposed a model for ubiquitin code in proteasomal degradation (Figure 3). In this model, the p97-Ufd1-Npl4 complex is the factor that is most upstream of the proteasome and is involved in recognizing Lys48-linked ubiquitin conjugates; in particular, Npl4 governs the Lys48 selectivity of proteasomal degradation. p97-dependent segregation may antagonize ubiquitylation and terminate ubiquitin chain elongation on the substrate complex. It can be hypothesized that Npl4 recognizes and segregates Lys48 chains (which are roughly heptamers in length) on the substrate. Rad23 and Dsk2 then capture the substrates and deliver them to the proteasome. Twomey et al. demonstrated that otu1, a p97-associated deubiquitylating enzyme, cleaves ubiquitin chains on the substrate and requires efficient substrate unfolding by p97. It remains unclear how deubiquitylated substrates are handed over to shuttle factors. Besides, the decrease in the segregation activity of mutant p97 results in the elongation of the ubiquitin chain on the substrates. It appears that substrates that remain in complexes can be transported to the proteasome and inhibit proteasome activity by competing with authentic ubiquitylated substrates.

## 4. Intracellular Dynamics of the Proteasome

### 4.1. Discovery of Stress-Dependent Proteasome Nuclear Foci

The proteasome is localized in both the cytoplasm and the nucleus. In particular, in proliferating yeast and mammalian cells, the proteasome is highly enriched in the nucleus and is presumably involved in the degradation of nuclear proteins [73,74,75]. When nuclear proteasomes are eliminated in yeast by a chemically-induced dimerization system, ubiquitylated proteins show significant accumulation [76]. However, the biological importance of proteasomal degradation in the nucleus under basal conditions is not well characterized. Abnormal proteins, such as those that are misfolded, are presumably constantly produced and then degraded by the proteasome, not only in the cytoplasm but also in the nucleus. During our microscopic analysis of the proteasome, we unexpectedly observed that proteasomes rapidly formed multiple foci in the nucleus upon hyperosmotic stress [36]. These proteasome foci were transient structures that emerged within a minute after hyperosmotic stimulation and disappeared after a few hours. Cryo-electron tomographic analysis successfully captured the clustering of proteasomes in the nucleus upon osmotic stimulation. We investigated whether these proteasome foci consisted of droplets, as they contained no aggregates or scaffold proteins. Indeed, these foci had droplet-like properties: they consisted of small foci that fused into larger foci, their shape was almost spherical, and they dissolved following the addition of 1,6-hexanediol, an aliphatic alcohol that destabilizes liquid droplets. Proteasome foci did not co-localize with known nuclear structures, such as promyelocytic leukemia bodies or Cajal bodies, suggesting that the nuclear proteasome foci induced by hyperosmotic stress have distinct roles. Liquid–liquid phase separation (LLPS) has become a hot topic in the life sciences in recent years. Cellular structures formed through LLPS are called membrane-less organelles or biomolecular condensates. These include stress granules, cytosolic p-bodies, and nuclear speckles [77]. LLPS is a rapid, reversible, and widespread compartmentalization mechanism in cells. Distinct subcompartments facilitate the spatiotemporal regulation of biological reactions [77]. We found that proteasome foci mainly co-localized with Lys48-linked ubiquitin chains and did not form in cells that were treated with the E1 inhibitor. By contrast, the number and size of foci increased and their clearance was delayed in cells treated with a proteasome inhibitor or p97 inhibitor. Thus, ubiquitylated substrates are required for the formation of proteasome foci and proteasomal degradation is necessary for their clearance, suggesting that proteasome foci are the proteolytic sites of ubiquitylated proteins. Proteomics analysis revealed that the ubiquitylation level of several housekeeping proteins, including histones, HSP90 (HSP90AB1 and HSP90AA1), and ribosomal proteins, increased under hyperosmotic stress, suggesting that this stress has global effects on the cellular proteome. In cultured cells, about 75,000 ribosome subunit molecules are synthesized per minute, and orphan subunits that fail to be incorporated into ribosomes are degraded by the proteasome [78,79,80]. Electron microscopic analysis showed that the nucleolar dense fibrillar compartment, where pre-ribosomes are assembled, was disrupted under hyperosmotic stress. Hyperosmotic stress also inhibited ribosome assembly in the nucleus, suggesting that the assembly process is susceptible to this condition. Some ribosomal proteins (RPL15, RPL29) aggregated in the nucleoplasm upon hyperosmotic stress and merged with proteasome foci, and they subsequently disappeared within an hour. These findings suggest that orphan ribosomal subunits are one of the major types of substrates in proteasome foci under hyperosmotic stress.

### 4.2. Molecular Mechanism of the Formation of Proteasome Foci

We also revealed that some proteasome-interacting proteins, including p97, RAD23B, and the ubiquitin ligase UBE3A (also known as E6-AP), co-localized to proteasome foci. The knockout of UBE3A reduced the number of proteasome foci formed upon hyperosmotic stress, suggesting that UBE3A regulates the proteasome itself or the ubiquitylation of substrate proteins in the foci [81], but the underlying molecular mechanism remains unknown. On the other hand, the proteasome foci were not detected in RAD23B knockout cells. We first assumed that the ubiquitylated proteins form foci without RAD23B. However, neither proteasomes nor ubiquitin formed foci in RAD23B knockout or mutated in the UBA domain cells, suggesting that the ubiquitin-binding ability of RAD23B induces the phase separation of ubiquitylated proteins. Proteins that induce LLPS can be classified into two groups. One consists of denatured proteins with intrinsically disordered regions (IDRs) such as fused in sarcoma (FUS) or tar DNA binding protein-43 (TDP-43); these proteins phase-separate with RNA [82,83]. Others cause phase separation due to the multivalent interaction of molecules which use multiple domains like the Src homology 3 (SH3) domain and the proline-rich motif [84]. RAD23B has two UBA domains, so it can bind to two ubiquitin chains. Each UBA domain weakly interacts with a ubiquitin monomer, specifically in a 1:1 molecular ratio, but it is known that the avidity increases exponentially when ubiquitin forms a chain [85]. We observed the co-phase separation of RAD23B and Lys48 chains in vitro. RAD23B prefers ubiquitin chains with four or more ubiquitin molecules, and both UBA domains of RAD23B are required for droplet formation. Therefore, the LLPS of proteasomes, ubiquitin chains, and RAD23B is mediated through multivalent interactions between polyubiquitin and RAD23B.

**Figure 4:**
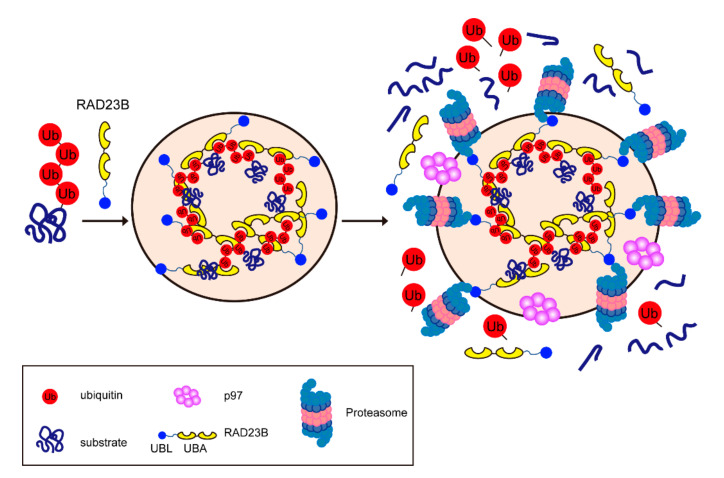
Hyperosmotic stress-induced proteasome foci in the nucleus. The multivalent interactions between polyubiquitin chains and the UBA of RAD23B drive the liquid–liquid phase separation (LLPS). Then, the proteasomes and p97 are recruited to the foci and degrade the ubiquitylated substrates.

Based on these findings, we proposed a model for the formation of proteasome foci (Figure 4). Hyperosmotic stress increases molecular crowding and accumulates ubiquitylated proteins such as ribosomal proteins. Consequently, RAD23B and ubiquitylated substrates form liquid droplets by LLPS, and proteasomes and their cofactors are recruited to the foci. We assume that the proteasome foci are the sites for the sequestration and efficient degradation of ubiquitylated proteins in the nucleus. Like RAD23B, UBQLN is also linked to LLPS, though it is a completely different mechanism. UBQLN2 is recruited to stress granules and undergoes LLPS without ubiquitin [86]. The LLPS is promoted by multivalent interactions across the IDR and the UBA domain of UBQLN2.

Interestingly, ubiquitin binding disrupts the UBQLN2 LLPS, suggesting that ubiquitin and UBQLN2 interactions result in the concomitant extraction of the polyubiquitylated substrate from stress granules. Importantly, some ALS-linked UBQLN mutations promote UBQLN2 oligomerization and accelerate LLPS formation in vitro [87], implying that the solidification of UBQLN2 directly links to ALS’ pathology. It has been reported that autophagy, another proteolytic system, also utilizes ubiquitin chain-dependent phase separation. The ubiquitin-selective autophagy adapter p62 has a Phox and Bem1 (PB1) domain, a UBA domain, and an LC3-binding domain for oligomer formation. p62 collects ubiquitylated substrates upon proteasome inhibition and oxidative stress, forms a droplet structure, and facilitates autophagy. The formation of these droplets (called p62 bodies) requires polyubiquitin chains linked with eight or more ubiquitins, and the UBA domain in p62 is important for the formation of p62 bodies [88]. Thus, one of the biological roles of polyubiquitin chain formation may be to induce LLPS. Since more than 100 proteins with UBDs are encoded in the human genome and many have multiple UBDs, a distinct LLPS mechanism is likely used by each ubiquitin-dependent pathway.

## 5. Conclusions and Perspective

Emerging evidence, reviewed in this article, emphasizes the crucial steps of substrate extraction/segregation and transport that are mediated by p97 and shuttling factors for proteasomal degradation. However, further studies are needed to elucidate how these steps together impact the ubiquitylated substrate to achieve effective degradation. In addition, the spatiotemporal and elaborate regulation of proteasomes is important for cell responses to the intra- and extracellular environment, since the proteasome plays a fundamental role in protein homeostasis. Since a number of proteasome-containing structures are known [89,90,91,92], the discovering of the osmotic stress-inducing proteasome phase separation sheds light on the importance of proteasome dynamics in response to the cellular environment. As the intracellular density of biomolecules is very high, the compartmentation of proteasomes and substrates with the help of shuttling factors may facilitate efficient proteasomal degradation. We expect that research over the next decade on the molecular mechanisms of substrate delivery and proteasome dynamics will contribute to a comprehensive understanding of the ubiquitin–proteasome system.
